# 2-Iodo-1,3-dimethoxy­benzene

**DOI:** 10.1107/S1600536809025264

**Published:** 2009-07-08

**Authors:** Li-Ping Xue, Jian-Hua Qin

**Affiliations:** a College of Chemistry and Chemical Engineering, Luoyang Normal University, Luoyang 471022, People’s Republic of China

## Abstract

Crystals of the title compound, C_8_H_9_IO_2_, were obtained from a dimethyl sulfoxide solution of 2,6-dimethoxy­benzoic acid and iodo­benzene diacetate under a nitro­gen atmosphere at 353 K. In the crystal structure, mol­ecules are linked by weak C—H⋯π inter­actions, generating inter­penetrating one-dimensional chains of perpendicularly oriented mol­ecules extending along [011] and [0
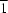
1]. Chains are also formed through non-bonding C—I⋯π contacts extending in the same directions, projecting a zigzag motif in view down [100]. The I⋯*Cg* distance is 3.695 (2) Å and the C—I⋯*Cg* angle is 164.17 (14)°. The mol­ecular symmetry *m* coincides with the mirror plane of the space group *Cmc*2_1_, resulting in a half-mol­ecule in the asymmetric unit (*Z*′ = ½).

## Related literature

For the development of a deca­rboxylative palladation reaction and its use in a Heck-type olefination of arene carboxyl­ates, see: Myers *et al.* (2002 ▶). For a novel system for deca­rboxylative bromination, see: Telvekar & Chettiar (2007 ▶). For related structures, see: Kirsop *et al.* (2004 ▶); Ali *et al.* (2008 ▶). For a database study of C-halogen–π inter­actions and their influence on mol­ecular conformation and crystal packing, see: Prasanna & Guru Row (2000 ▶). For structure validation in chemical crystallography, see: Spek (2009 ▶).
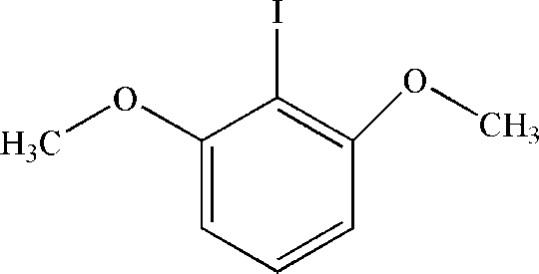


## Experimental

### 

#### Crystal data

C_8_H_9_IO_2_
*M* *_r_* = 264.05
Orthorhombic, 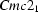

*a* = 12.5767 (13) Å
*b* = 8.6788 (8) Å
*c* = 8.4338 (9) Å
*V* = 920.55 (16) Å^3^
*Z* = 4
Mo *K*α radiation
μ = 3.43 mm^−1^
*T* = 296 K
0.23 × 0.19 × 0.16 mm

#### Data collection

Bruker P4 diffractometer
Absorption correction: multi-scan (*SADABS*; Bruker, 1997 ▶) *T*_min_ = 0.500, *T*_max_ = 0.616 (expected range = 0.469–0.578)
2731 measured reflections
850 independent reflections
840 reflections with *I* > 2σ(*I*)
*R*_int_ = 0.017

#### Refinement

*R*[*F* ^2^ > 2σ(*F* ^2^)] = 0.019
*wR*(*F* ^2^) = 0.046
*S* = 1.12
850 reflections
55 parameters
1 restraint
H-atom parameters constrained
Δρ_max_ = 1.02 e Å^−3^
Δρ_min_ = −0.84 e Å^−3^
Absolute structure: Flack (1983 ▶), 362 Friedel pairs
Flack parameter: −0.05 (4)

### 

Data collection: *SMART* (Bruker, 1997 ▶); cell refinement: *SAINT* (Bruker, 1997 ▶); data reduction: *SAINT*; program(s) used to solve structure: *SHELXS97* (Sheldrick, 2008 ▶); program(s) used to refine structure: *SHELXL97* (Sheldrick, 2008 ▶); molecular graphics: *SHELXTL* (Sheldrick, 2008 ▶); software used to prepare material for publication: *SHELXTL* and *PLATON* (Spek, 2009 ▶).

## Supplementary Material

Crystal structure: contains datablocks I, global. DOI: 10.1107/S1600536809025264/si2185sup1.cif

Structure factors: contains datablocks I. DOI: 10.1107/S1600536809025264/si2185Isup2.hkl

Additional supplementary materials:  crystallographic information; 3D view; checkCIF report

## Figures and Tables

**Table 1:** Hydrogen-bond geometry (Å, °)

*D*—H⋯*A*	*D*—H	H⋯*A*	*D*⋯*A*	*D*—H⋯*A*
C1—H1*A*⋯*Cg*1^i^	0.93	2.94	3.824 (9)	159

Symmetry code: (i) 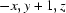. *Cg*1 is the centroid of the C1–C4/C3*A*/C2*A* ring.

**Fig. 1.**
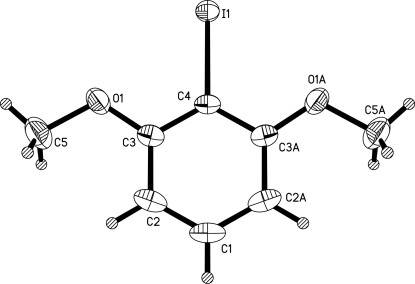
The molecular structure of the title compound. Symmetry code: (2 - x, y, z). Displacement ellipsoids for non-hydrogen atoms are drawn at the 30% probability level.

**Fig. 2.**
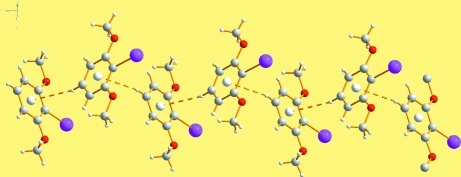
A view of the one-dimensional weak C—H···π contacts in the title compound.

**Fig. 3.**
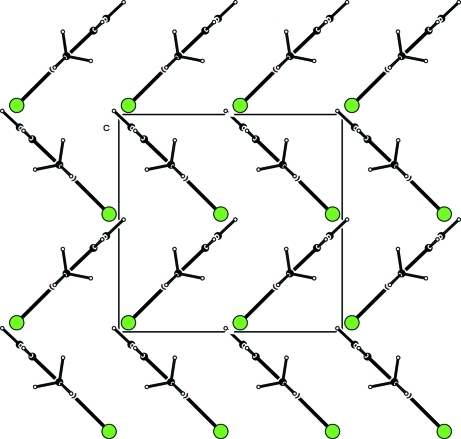
A view down the a axis showing a section of the zigzag motif of the title compound.

## supplementary crystallographic information

### Comment

Decarboxylation arene carboxylic acids accompanied by simultaneous replacement with different function groups is a useful reaction in organic chemistry (Myers *et al.*, 2002;). Especially iodobenzene derivatives have been found widespread application in organic synthesis because of their selectivity and simplicity of use (Telvekar & Chettiar, 2007). Recently, we found iodobenzene derivatives could be formed by arene carboxylic acid with reaction of PhI(OAc)_2_. As part of our studies, we report herein the synthesis and crystal structure of the title compound (Fig. 1). The asymmetric unit of the cell contains a half-molecule (Z' = 1/2), which is completed by the space group symmetry *m*. Atoms I1, C4, C1, H1A occupy the special positions in the mirror plane *m*. The bond length of C4—I1 is 2.090 (5) Å. The two I—C—C angles, related by mirror symmetry, are 119.5 (2)°.

The molecules in the crystal structure are linked by weak C—H···π interactions to generate a one-dimensional supramolecular structure (Fig. 2). The bond length of C1—H1A···*Cg*1 is 3.824 (9) Å (Table. 1), *Cg*1 is the centroid of the C1 C2 C3 C4 C3A C2A ring. In a CSD database study, Prasanna & Guru Row (2000) reported about C-halogen···π interactions and their influence on molecular conformation and crystal packing. The authors found 171 intermolecular C—I···π contacts in the literature, with a mean I···C_(__π__-system)_ atomic distance of 3.698 (13) Å. In the course of the structure validation (Spek, 2009) of the title compound, a similar geometric parameter (I1···Cg1^ii^ = 3.695 (2) Å) has been found. The C4···Cg1^ii^ distance amounts to 5.735 (5) Å, and the angle C4—I1···Cg^ii^ is 164.17 (14) Å. Symmetry code: (ii = -*x*, *y* + 2, *z* - 1). The C4—H1A···π and nonbonding C4—I1···π contacts generate interpenetrating one-dimensional chains of perpendicularly oriented molecules extending along the [0 1 1] and [0 1 1] directions, projecting a zigzag motif in view down [1 0 0] (Fig.3).

### Experimental

The title compound was obtained from a mixture of 2,6-Dimethoxybenzoic acid (36 mg) with Iodobenzene diacetate (77 mg) in DMSO (2 ml) under a nitrogen atmosphere at 353 K for 24 h. The crude product was isolated and purified by silica gel column chromatography. Colorless prism-shaped crystals of (I) suitable for X-ray diffraction were grown by slow evaporation of a dichloromethane solution at room temperature.

### Refinement

All hydrogen atoms were positioned geometrically and treated as riding, with C—H = 0.93 Å (CH) and *U*_iso_(H) = 1.2*U*_eq_(C), and with C—H = 0.96 Å (CH3) and *U*_iso_(H) = 1.5*U*_eq_(C).

### Crystal data

**Table:**

C_8_H_9_IO_2_	*F*(000) = 504
*M**_r_* = 264.05	*D*_x_ = 1.905 Mg m^−^^3^
Orthorhombic, *C**m**c*2_1_	Mo *K*α radiation, λ = 0.71073 Å
Hall symbol: C 2c -2	Cell parameters from 1365 reflections
*a* = 12.5767 (13) Å	θ = 3.7–27.5°
*b* = 8.6788 (8) Å	µ = 3.43 mm^−^^1^
*c* = 8.4338 (9) Å	*T* = 296 K
*V* = 920.55 (16) Å^3^	Prism, white
*Z* = 4	0.23 × 0.19 × 0.16 mm

### Data collection

**Table:**

Bruker P4 diffractometer	850 independent reflections
Radiation source: fine-focus sealed tube	840 reflections with *I* > 2σ(*I*)
graphite	*R*_int_ = 0.017
ω scans	θ_max_ = 25.5°, θ_min_ = 3.7°
Absorption correction: multi-scan (*SADABS*; Bruker, 1997)	*h* = −15→15
*T*_min_ = 0.500, *T*_max_ = 0.616	*k* = −10→7
2731 measured reflections	*l* = −9→10

### Refinement

**Table:**

Refinement on *F*^2^	Secondary atom site location: difference Fourier map
Least-squares matrix: full	Hydrogen site location: inferred from neighbouring sites
*R*[*F*^2^ > 2σ(*F*^2^)] = 0.019	H-atom parameters constrained
*wR*(*F*^2^) = 0.046	*w* = 1/[σ^2^(*F*_o_^2^) + (0.0245*P*)^2^ + 0.6278*P*] where *P* = (*F*_o_^2^ + 2*F*_c_^2^)/3
*S* = 1.12	(Δ/σ)_max_ = 0.001
850 reflections	Δρ_max_ = 1.02 e Å^−^^3^
55 parameters	Δρ_min_ = −0.84 e Å^−^^3^
1 restraint	Absolute structure: Flack (1983), 362 Friedel pairs
Primary atom site location: structure-invariant direct methods	Flack parameter: −0.05 (4)

### Fractional atomic coordinates and isotropic or equivalent isotropic displacement parameters (Å^2^)

**Table:**

	*x*	*y*	*z*	*U*_iso_*/*U*_eq_	
I1	1.0000	0.95668 (3)	0.54168 (9)	0.04533 (12)	
O1	0.8137 (2)	0.7922 (4)	0.7118 (3)	0.0562 (8)	
C1	1.0000	0.5564 (7)	0.9408 (11)	0.073 (2)	
H1A	1.0000	0.4784	1.0163	0.088*	
C2	0.9046 (4)	0.6130 (5)	0.8860 (6)	0.0637 (12)	
H2A	0.8409	0.5739	0.9250	0.076*	
C3	0.9038 (3)	0.7287 (4)	0.7725 (4)	0.0432 (9)	
C4	1.0000	0.7859 (5)	0.7165 (6)	0.0374 (11)	
C5	0.7137 (4)	0.7340 (7)	0.7685 (7)	0.0776 (16)	
H5A	0.6566	0.7868	0.7160	0.116*	
H5B	0.7087	0.7505	0.8808	0.116*	
H5C	0.7091	0.6256	0.7464	0.116*	

### Atomic displacement parameters (Å^2^)

**Table:**

	*U*^11^	*U*^22^	*U*^33^	*U*^12^	*U*^13^	*U*^23^
I1	0.04655 (17)	0.05029 (18)	0.0391 (2)	0.000	0.000	0.01194 (17)
O1	0.0454 (16)	0.0700 (18)	0.0531 (19)	−0.0124 (14)	0.0033 (14)	0.0111 (15)
C1	0.099 (6)	0.052 (4)	0.068 (5)	0.000	0.000	0.030 (3)
C2	0.085 (3)	0.051 (2)	0.055 (3)	−0.016 (2)	0.009 (2)	0.014 (2)
C3	0.058 (2)	0.0384 (16)	0.033 (2)	−0.0055 (16)	0.0005 (16)	−0.0015 (15)
C4	0.055 (3)	0.030 (2)	0.027 (3)	0.000	0.000	−0.0008 (19)
C5	0.056 (3)	0.086 (4)	0.091 (5)	−0.023 (3)	0.010 (3)	0.007 (3)

### Geometric parameters (Å, °)

**Table:**

I1—C4	2.090 (5)	C2—H2A	0.9300
O1—C3	1.359 (5)	C3—C4	1.391 (5)
O1—C5	1.437 (6)	C4—C3^i^	1.391 (5)
C1—C2	1.376 (6)	C5—H5A	0.9600
C1—C2^i^	1.376 (6)	C5—H5B	0.9600
C1—H1A	0.9300	C5—H5C	0.9600
C2—C3	1.388 (5)		
			
C3—O1—C5	117.5 (4)	C3^i^—C4—C3	121.0 (5)
C2—C1—C2^i^	121.3 (6)	C3^i^—C4—I1	119.5 (2)
C2—C1—H1A	119.3	C3—C4—I1	119.5 (2)
C2^i^—C1—H1A	119.3	O1—C5—H5A	109.5
C1—C2—C3	119.8 (5)	O1—C5—H5B	109.5
C1—C2—H2A	120.1	H5A—C5—H5B	109.5
C3—C2—H2A	120.1	O1—C5—H5C	109.5
O1—C3—C2	124.0 (4)	H5A—C5—H5C	109.5
O1—C3—C4	116.9 (3)	H5B—C5—H5C	109.5
C2—C3—C4	119.1 (4)		
			
C2^i^—C1—C2—C3	0.5 (11)	O1—C3—C4—C3^i^	179.6 (3)
C5—O1—C3—C2	−0.7 (6)	C2—C3—C4—C3^i^	0.1 (7)
C5—O1—C3—C4	179.9 (4)	O1—C3—C4—I1	−1.9 (5)
C1—C2—C3—O1	−179.7 (5)	C2—C3—C4—I1	178.6 (3)
C1—C2—C3—C4	−0.3 (7)		

Symmetry codes: (i) −*x*+2, *y*, *z*.

### Hydrogen-bond geometry (Å, °)

**Table:**

*D*—H···*A*	*D*—H	H···*A*	*D*···*A*	*D*—H···*A*
C1—H1A···Cg1^ii^	0.93	2.94	3.824 (9)	159

Symmetry codes: (ii) −*x*, *y*+1, *z*.
